# Traditional Chinese medicine in diabetic kidney disease: multifaceted therapeutic mechanisms and research progress

**DOI:** 10.1186/s13020-025-01150-w

**Published:** 2025-07-01

**Authors:** Rushuang Cai, Chunying Li, Yong Zhao, Haisheng Yuan, Xiaomeng Zhang, Aihua Liang, Yan Yi

**Affiliations:** State Key Laboratory for Quality Ensurance and Sustainable use of Dao-Di Herbs, Institute of Chinese Materia Medica, China Academy of Chinese Medical Sciences, Beijing, 100700 China

**Keywords:** Diabetic kidney disease, Traditional Chinese medicine, Pharmacological mechanism

## Abstract

Diabetic kidney disease (DKD) is a severe complication of diabetes and a leading cause of end-stage renal disease (ESRD). It is characterized by a complex pathogenesis that involves genetic, epigenetic, metabolic, and multifactorial elements. Current therapeutic approaches for DKD have significant limitations. In contrast, Traditional Chinese Medicine (TCM), with its personalized treatment based on syndrome differentiation, shows potential to modulate multiple pathological pathways implicated in DKD. This review provides a detailed examination of DKD pathogenesis and the progress in TCM research, offering valuable insights for DKD treatment research and clinical practice.

## Introduction

DKD, a prevalent and severe microvascular complication of diabetes, has exhibited a significant rise in global incidence. Epidemiological investigations reveal that the prevalence of DKD among individuals with diabetes is considerable, impacting nearly half of this population globally. DKD has emerged as a leading cause of chronic kidney disease (CKD) and ESKD [1, 2], thereby exerting a substantial burden on patient health and quality of life and placing considerable strain on societal medical resources. According to data from the International Diabetes Federation, the number of diabetic patients aged 20–79 has reached 537 million and is projected to escalate to 784 million by 2045, with at least 40% of these individuals at risk of developing CKD [3]. The etiology of DKD is multifactorial, characterized by polygenic inheritance, familial aggregation, and ethnic disparities. Clinically, DKD is marked by a decline in glomerular filtration rate (GFR), persistent proteinuria, and hypertension. Pathologically, it is distinguished by thickening of the glomerular basement membrane (GBM), accumulation of extracellular matrix (ECM), and podocyte injury [4].

Contemporary management of DKD primarily centers on hemodynamic optimization, glycemic control, blood pressure management, lipid regulation, and the administration of renin–angiotensin–aldosterone system (RAAS) inhibitors. RAAS inhibitors, sodium-glucose cotransporter 2 (SGLT2) inhibitors, and dipeptidyl peptidase-4 (DPP-4) inhibitors are frequently employed [5, 6]. Moreover, emerging therapeutic modalities, such as protein kinase C (PKC) inhibitors, mineralocorticoid receptor antagonists (MRAs), and endothelin receptor (ETR) antagonists, have shown favorable outcomes in preclinical and clinical investigations [7]. However, these therapeutic strategies are not without limitations. For example, SGLT2 inhibitors may heighten the risk of genitourinary tract infections and precipitate adverse events associated with volume depletion [8]. The long-term renal effects of DPP-4 inhibitors remain to be fully elucidated. The clinical utility of MRAs is constrained by their propensity to induce hyperkalemia and other adverse effects [9]. Despite their capacity to somewhat retard disease progression, these treatments often fall short of meeting clinical demands due to the intricate pathogenesis of DKD.

Against this backdrop, TCM exhibits distinct advantages in the management of DKD. TCM underscores personalized therapy, which entails devising treatment plans based on comprehensive syndrome differentiation encompassing the patient’s constitution, symptoms, tongue signs, and pulse conditions. By modulating metabolic abnormalities and enhancing microcirculatory function in both renal tissues and systemic circulation, TCM can effectively reduce urinary protein levels, thereby safeguarding renal function.

## Pathological mechanisms of DKD

### Genetic susceptibility and epigenetic factors

Genetic predisposition represents a critical determinant in the pathogenesis of DKD among individuals with diabetes mellitus [10, 11]. The heritable nature of DKD is supported by familial clustering patterns observed in nephropathy cohorts. Single nucleotide polymorphisms (SNPs), which represent the predominant form of genomic variation, demonstrate significant associations with DKD susceptibility. Mechanistic studies have identified disease-associated loci, including CD28-rs3116494 and CD80-rs3850890 [12]. Genome-wide association studies have identified pathogenic variants in collagen type IV alpha 3 chain (COL4A3), a gene encoding critical structural components of the glomerular filtration barrier [13–15]. These findings highlight the complex interplay between polygenic inheritance and environmental exposures in the development of DKD.

Hyperglycemia initiates metabolic disturbances and epigenetic alterations, including DNA methylation and the regulation of non-coding RNAs, such as microRNAs (miRNAs) and long non-coding RNAs (lncRNAs), which significantly influence DKD [16]. MiRNAs, which consist of 19–24 nucleotide single-stranded RNA molecules, play a critical role in fibrotic processes associated with DKD. Notable miRNA families include miR-192, miR-21, miR-200, and miR-29 [17]. For example, transforming growth factor-beta (TGF-β) induces the expression of miR-216a and miR-217, which target phosphate and tension homology deleted on chromosome ten (PTEN) and activate the Phosphatidylinositol 3-Kinase/Protein Kinase B (PI3K/Akt) signaling pathway in mesangial cells [18]. In type 2 diabetic mice, elevated levels of miR-21 correlate with decreased PTEN and Smad7 expression alongside increased TGF-β1, Smad3, and nuclear factor kappa-B (NF-κB) activity; this promotes mesangial cell fibrosis and hypertrophy [19]. Additionally, miRNAs modulate ECM protein accumulation by regulating collagen gene expression [20]. LncRNAs are longer than 200 nucleotides and interact with proteins, RNA molecules, and DNA to regulate gene expression as well as various cellular processes [21, 22]. Taurine up-regulated gene 1 located on chromosome 22q12-regulates mitochondrial function in podocytes through peroxisome proliferator-activated receptor gamma coactivator 1-alpha (PGC-1α) signaling pathways that improve diabetes-induced CKD in murine models [23]. Other lncRNAs such as metastasis associated lung adenocarcinoma transcript 1 (MALAT1), plasmacytoma variant translocation 1 (PVT1), antisense noncoding RNA gene at the INK4 locus (ANRIL), and Erb-B2 receptor tyrosine kinase 4-immunoreactivity (Erbb4-IR) have been implicated in the modulation of ECM proteins and profibrotic factors within DKD. Importantly, MALAT1 also regulates inflammatory genes and cytokines that link inflammation to DKD pathogenesis [24].

### Metabolic factors

In the pathogenesis of DKD, multiple mechanisms act in concert, with oxidative stress being a pivotal factor. AGEs are formed from macromolecules such as proteins and lipids through non-enzymatic glycation, with both endogenous and exogenous origins. Under normal conditions, the production and clearance of AGEs are balanced. However, in DKD, hyperglycemia leads to the accumulation of AGEs. AGEs damage tissues by cross-linking ECM proteins and interacting with cell-surface receptors [25–27]. PKC, a member of the serine/threonine kinase family, is activated under chronic hyperglycemic conditions due to elevated diacylglycerol accumulation. Once activated, PKC promotes the synthesis of type IV collagen and fibronectin, thickening the GBM and inducing interstitial fibrosis. PKC also affects angiotensin-II-mediated vasoregulation, causing glomerular hyperfiltration, and influences vascular permeability and neovascularization by regulating vascular endothelial growth factor (VEGF) expression, thereby accelerating proteinuria progression [28–30]. The polyol pathway, an alternative route of glucose metabolism, experiences over-activation of aldose reductase (AR) due to hyperglycemia in DKD, leading to substantial intracellular sorbitol accumulation. This process damages the kidneys by generating AGEs, reactive oxygen species (ROS), and activating the TGF-β signaling pathway, thereby disrupting renal oxidative stress, fibrosis, and epithelial-mesenchymal transition (EMT) [31, 32]. Notably, oxidative stress is a key pathogenic factor in DKD progression. Hyperglycemia activates multiple pathways, including the polyol pathway, PKC, AGEs/RAGE, and hexosamine, causing excessive ROS accumulation. This disrupts the intracellular oxidative stress balance, triggering renal endothelial cell apoptosis, inflammation, autophagy dysregulation, and fibrosis, ultimately severely impairing renal function [33, 34].

### Hemodynamic factors

Sustained hyperglycemia increases the GFR and, in conjunction with hypertension, leads to renal hyperperfusion. This results in mesangial matrix expansion and thickening of the basement membrane, potentially progressing to focal glomerulosclerosis. In early diabetic models, the release of vasodilatory mediators, including nitric oxide (NO), is inhibited, thereby affecting afferent arteriolar dilation [35, 36]. Hyperglycemia also upregulates SGLT2 in renal tubules, increasing glucose and sodium reabsorption and reducing macula densa sodium content. This leads to afferent arteriole dilation and an increased filtration rate through tubuloglomerular feedback [37]. Overactivation of the RAAS significantly contributes to systemic hypertension in DKD. Angiotensin II interacts with NO in renal cells, influencing organ damage or protection [38, 39]. Endothelin-1 promotes proteinuria and is associated with renal inflammation and fibrosis, playing a role in hypertension and renal injury [40, 41]. Hemodynamic overload not only exacerbates glomerular injury but also synergizes with inflammatory cascades to promote renal fibrosis. The subsequent section explores the central role of the nucleotide-binding oligomerization domain-like receptor family pyrin domain-containing 3 (NLRP3) inflammasome and TGF-β signaling in this process.

### Inflammation and fibrosis factors

DKD is characterized by a chronic, low-grade inflammatory state primarily driven by innate immune activation. Central to this process is the NLRP3 inflammasome, which serves as a pivotal mediator of pro-inflammatory cytokine activation in the pathophysiology of DKD [42]. The NLRP3 inflammasome, a multi-protein complex comprising NLRP3, apoptosis-associated speck-like protein containing a CARD domain (ASC), and pro-caspase-1, is activated under hyperglycemic conditions. This activation leads to the cleavage and activation of caspase-1, which in turn activates interleukin-1 beta (IL-1β) and IL-18. These cytokines further amplify the inflammatory response and renal damage [43, 44]. In the pathogenesis of DKD, C-reactive protein (CRP), nuclear receptor subfamily 4 group A member 1 (NR4A1), and the high glucose-activated inositol requiring enzyme 1 alpha/spliced X-box binding protein 1 (IRE1α/sXBP1) pathway are intricately interconnected. Collectively, they drive renal injury and fibrosis through the activation of the NLRP3 inflammasome. Mechanistically, CRP promotes DKD progression by activating the NLRP3 inflammasome via a Smad3-mediated pathway [45]. Under DKD conditions, NR4A1 upregulation simultaneously triggers the activation of both the NLRP3 inflammasome and the PI3K/AKT pathway, thereby exacerbating renal fibrosis [46]. Concurrently, high glucose levels induce renal tubular injury by activating the NLRP3 inflammasome through the IRE1α/sXBP1 signaling axis [47].

The TGF-β1 signaling pathway is central to diabetic renal fibrosis, influencing kidney function by promoting ECM expression and inducing dedifferentiation of renal cells [48]. Connective Tissue Growth Factor (CTGF), which is upregulated in glomerulonephritis, regulates the expression of monocyte chemoattractant protein-1 (MCP-1) and facilitates ECM deposition, culminating in glomerular damage and fibrosis [49, 50].

### Autophagy

Autophagy, a lysosomal degradation pathway, is essential for maintaining the homeostasis of glomeruli and renal tubules and is regulated by signaling pathways, including the mammalian target of rapamycin (mTOR), AMP-activated protein kinase (AMPK), and silent information regulator T1 (SIRT1) [51]. The mTOR complex plays a key role in cellular growth and metabolism, and under hyperglycemic conditions, increased activity of mammalian target of rapamycin complex 1 (mTORC1) may suppress autophagy, leading to renal cell injury and the progression of DKD [52, 53]. Smad3 inhibits lysosomal biogenesis by suppressing transcription factor EB (TFEB), resulting in lysosomal depletion and impaired autophagic flux, which exacerbates renal tubular epithelial cell injury [54].

### Exosomes

Exosomes, nanoscale extracellular vesicles approximately 40–160 nm in diameter, are secreted by various cell types and play a significant role in intercellular communication and the pathogenesis of diseases, including DKD [55, 56]. These vesicles can transport proteins, metabolites, and nucleic acids, thereby modulating disease progression. Exosomes are implicated in DKD through mechanisms involving inflammation, autophagy, and insulin resistance [57]. Macrophages, which are central to inflammatory processes, can accelerate DKD progression by promoting the release of TGF-β, ROS, VEGF, and cytokines [58]. M1 macrophages secrete pro-inflammatory mediators, while M2 macrophages are associated with fibrotic processes [59]. Hyperglycemia activates macrophages via pathways involving TGF-β1, MCP-1, tumor necrosis factor-alpha (TNF-α), and IL-1β, leading to the upregulation of NF-κB and subsequent renal injury [60–62]. Glomerular endothelial cells (GECs), which contribute to DKD pathogenesis, can secrete exosomes that transfer circular RNA or mRNA to mesangial cells, thereby promoting renal fibrosis [63]. SIRT1, a regulator of glucose metabolism, has been linked to exosome secretion. Studies have shown that autophagy defects in Ad-Sirt1 mice increase exosome release, thereby affecting obesity and insulin resistance [64].

### Mitochondrial dynamics imbalance

Podocyte injury in DKD is closely associated with mitochondrial dysfunction, which, through the excessive generation of ROS, disrupts energy metabolism and increases oxidative stress, ultimately leading to apoptosis and subsequent podocyte damage [65]. The imbalance in mitochondrial dynamics, characterized by the dysregulation of fusion and fission processes, further exacerbates podocyte injury. Consequently, inhibiting mitochondrial fission can protect podocytes from albumin-induced cellular injury in DKD [66]. TCMs capable of modulating the imbalance of mitochondrial dynamics offer novel therapeutic avenues for delaying the progression of DKD.

### Pyroptosis

**Fig. 1:**
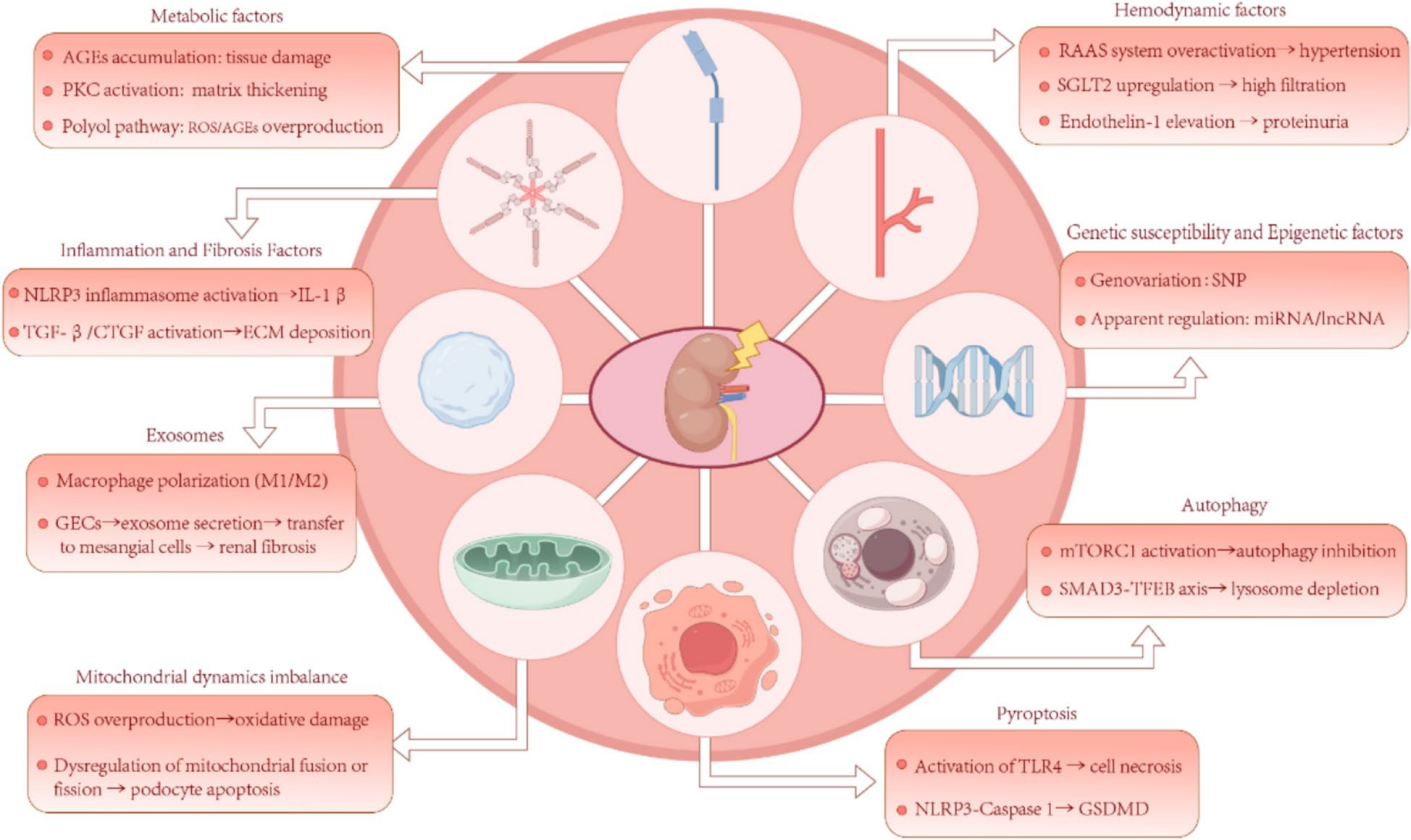
Pathological mechanism diagram of DKD (by Figdraw2.0). The arrow (→) indicates causation

Pyroptosis, a form of programmed cell death, is intimately associated with the activation of caspase-1 and the NLRP3 inflammasome. Under the influence of various external stimuli, the Gasdermin D (GSDMD) protein forms pores, leading to the disruption of cellular membrane integrity, which in turn triggers cell rupture, necrosis, and the release of cellular contents [67]. GSDMD-mediated pyroptosis plays a critical role in the onset and progression of DKD. Studies have indicated that caspase-11/4 and GSDMD-mediated pyroptosis are activated and participate in the loss of podocytes and the development of DKD under hyperglycemic conditions [68]. Furthermore, Yuan et al. have discovered that Gasdermin D is involved in the transition from apoptosis to pyroptosis in toll-like receptor 4 (TLR4)-mediated renal tubular epithelial cell injury in DKD (Fig. 1) [69].

The intricate interplay of genetic susceptibility, metabolic dysregulation, inflammation, and cellular dysfunction underscores the multifaceted nature of DKD pathogenesis. These mechanisms collectively drive renal fibrosis, podocyte loss, and progressive functional decline, posing significant challenges to conventional single-target therapies. In this context, TCM emerges as a promising therapeutic paradigm by integrating syndrome differentiation with multi-pathway modulation. The following sections elaborate on TCM’s typological treatment strategies and their alignment with the pathophysiological complexity of DKD.

## Typological treatment and research advancements of TCM formulas for DKD

TCM demonstrates unique advantages in managing DKD through its holistic philosophy and syndrome differentiation. Its multi-level, multi-target intervention aligns with the dynamic progression of DKD, which is characterized by root deficiencies (spleen-kidney Qi-Yin deficiency) and surface manifestations (damp-heat, blood stasis, turbid toxins). Modern studies confirm a triphasic trajectory: initial metabolic-inflammatory imbalances stemming from spleen-kidney deficiency, followed by fibrotic remodeling and toxin retention.

Therapeutic strategies integrate triple energizer modulation with stage-specific interventions. Upper energizer treatments replenish Qi and nourish Yin; middle energizer approaches strengthen the spleen and resolve dampness; lower energizer therapies tonify the kidneys. Early-stage management activates collaterals, mid-stage interventions combine reinforcement with pathogen removal, and late-stage protocols clear toxins. Collectively, these efforts improve metabolism, inhibit oxidative-inflammatory damage, and protect renal function.

Evidence supports TCM's multi-target regulatory potential in DKD. Randomized controlled trials (RCTs) have demonstrated significant reductions in proteinuria and stabilization of renal function, with meta-analyses confirming TCM's superiority over placebo [70]. Mechanistic studies reveal that herbal compounds modulate key pathways involved in inflammation, fibrosis, oxidative stress, and podocyte protection [71]. Further rigorous clinical and translational research is needed to fully explore the therapeutic potential of TCM in the management of DKD.

### Qi and Yin deficiency with blood stasis pattern DKD

In TCM, the treatment of diabetes with qi-yin deficiency primarily focuses on invigorating qi, strengthening the spleen, nourishing yin, and tonifying the kidney. Shenqi Dihuang Decoction (SQDHD) has demonstrated a significant therapeutic effect in patients with early-stage DKD characterized by qi-yin deficiency. In this formula, ginseng and astragalus are utilized to invigorate qi and strengthen the spleen; *rehmannia glutinosa*, *Chinese yam*, and *dogwood fruit* are employed to nourish yin and tonify the kidney; *alisma orientale* and *poria cocos* are included to promote diuresis and resolve dampness; and moutan cortex is added to clear heat and cool the blood.

Clinical trials have demonstrated that SQDHD effectively alleviates symptoms associated with DKD, such as fatigue, xerostomia, palmar-plantar hyperthermia, and lumbar weakness. Concurrently, SQDHD has been shown to improve glycemic control, attenuate renal injury markers, suppress pro-inflammatory cytokines, and augment antioxidant enzyme activities [72]. Experimental research suggests that the therapeutic mechanism of SQDHD may involve inhibiting arachidonic acid metabolism-associated ferroptosis via modulation of the acyl-coA synthetase long chain family member 4 (ACSL4)/lysophosphatidylcholine acyltransferase 3 (LPCAT3)/arachidonate 15-lipoxygenase (ALOX15) axis [73]. A meta-analysis of 13 RCTs further confirmed its efficacy in significantly reducing proteinuria, blood urea nitrogen (BUN), serum creatinine (Scr), and TCM syndrome scores in patients with stage III-IV DKD characterized by qi-yin deficiency [74]. Although adverse events were rarely reported across studies, indicating a favorable safety profile, the specific nature and incidence of potential side effects require further investigation. Additionally, the combination of SQDHD and acupoint injection has been shown to enhance efficacy in improving renal function and metabolic parameters in DKD patients with qi-yin deficiency and blood stasis patterns, with good tolerability observed in clinical trials [75].

Furthermore, Keluoxin Capsule (KLXC), an effective TCM preparation, functions to replenish qi and nourish yin, as well as promote blood circulation to remove blood stasis. It is applicable to cases of DKD characterized by qi-yin deficiency accompanied by blood stasis. Clinical research indicates that the combination of KLXC and Western medicine (such as Candesartan, Captopril) has a positive impact on DKD [76]. Specifically, it can reduce endoplasmic reticulum stress by inhibiting the activation of the protein kinase R-like endoplasmic reticulum kinase/activating transcription factor 4/DNA damage-inducible transcript 3 (PERK-ATF4-CHOP) signaling pathway [77]. Additionally, it can enhance the autophagic activity of podocytes and mitigate podocyte damage [78].

### Spleen-kidney Yang deficiency pattern DKD

In TCM theory, the treatment of diabetic patients with spleen-kidney yang deficiency syndrome primarily focuses on tonifying the spleen and kidney, warming yang, and promoting diuresis. Zhenwu Decoction (ZWD) is a classic TCM formula that embodies these therapeutic principles. Aconite and Poria in ZWD are known for their warming yang and diuretic effects [79]. *Ginger* and *Atractylodes* invigorate the spleen and dry dampness, while *Paeony* relieves spasms and pain. Consequently, ZWD is well-suited for treating diabetes with spleen-kidney yang deficiency [80]. A meta-analysis of 13 RCTs revealed that combining ZWD with conventional Western medicine (CWM) significantly enhances therapeutic outcomes in the management of DKD. Compared with CWM alone, the ZWD-CWM combination demonstrated superior clinical efficacy, as evidenced by significant reductions in fasting blood glucose, proteinuria, Scr, and BUN levels [81]. Further clinical studies have corroborated these findings, showing that modified ZWD combined with CWM not only reduces 24-h urinary protein levels and renal injury biomarkers (such as kidney injury molecule-1 and cystatin C) but also achieves a higher overall clinical efficacy rate compared with the control group. Moreover, the incidence of adverse reactions, such as dizziness and diarrhea, was comparable between the treatment and control groups [82].

Modern research indicates that ZWD effectively alleviates clinical symptoms such as fatigue, edema, and lumbago in patients, reduces urinary protein levels, lowers the incidence of ESKD, and improves long-term prognosis and survival outcomes [83]. The kidney-protective effects of ZWD may be attributed to its ability to ameliorate oxidative damage and energy metabolism disorders [84]. Specifically, ZWD promotes mitochondrial bioenergetics by activating nuclear factor erythroid 2-related factor 2 (Nrf2) and mitochondrial transcription factor A (TFAM) in renal tubules, thereby inhibiting renal fibrosis [85]. Additionally, ZWD has been shown to improve the general condition and renal function of db/db mice with spleen-kidney yang deficiency syndrome, reduce oxidative damage, and alleviate renal pathological changes. The underlying mechanism may involve modulating the Nrf2/Heme oxygenase-1 (HO-1)/Glutathione peroxidase 4 (GPX4) pathway [86].

Jingui Shenqi Pill (JGSQW) is a classic Chinese patent medicine known for its effects of warming and tonifying kidney yang, as well as promoting qi transformation and diuresis. It is indicated for various symptoms associated with kidney yang deficiency. Studies have demonstrated that JGSQW can reduce the 24-h urinary protein excretion rate in patients, alleviate edema, and decrease levels of Scr and BUN in patients with DKD, thereby protecting renal function [87]. The combination of JGSQW and metformin may exert synergistic effects by downregulating the expression of immune complex major histocompatibility complex (MHC class II) molecules and weakening the antigen presentation of MHC class II to cluster of differentiation 4 (CD4), thus alleviating renal fibrosis in patients with DKD [88].

### Liver-Kidney Yin deficiency pattern DKD

Liuwei Dihuang Pill (LWDHP) is a TCM formula applicable to individuals with kidney yin deficiency. In this formula, *Rehmannia glutinosa* serves to nourish kidney yin; *Dioscorea opposita* and *Cornus officinalis* function to tonify the liver, spleen, and kidney; Alisma orientale, Poria cocos, and Moutan cortex are included to clear and purge the deficient fire in the liver and kidney, as well as to promote diuresis and percolate dampness [89].

LWDHP improves insulin resistance and reduces blood glucose levels by nourishing kidney yin. Moreover, it regulates blood lipids, decreases lipid deposition in the kidneys, improves renal lipid metabolism, and alleviates lipid-toxicity-induced damage to the kidneys [90]. Research has demonstrated that LWDHP protects renal function through multiple pathways. For instance, it reduces levels of Scr, BUN, and 24-h urinary protein, and inhibits the expression of TGF-β, alpha-smooth muscle actin (α-SMA), Smad2, and Smad3, thereby ameliorating renal fibrosis in DKD [91].

### Yin deficiency with dryness-heat pattern DKD

The Zhibai Dihuang Pill (ZBDHP) is a traditional formula in which *Anemarrhena asphodeloides* and Cortex *Phellodendri* function to clear heat and purge fire, while *Rehmannia glutinosa*, Dioscorea *opposita*, and *Cornus* officinalis are employed to nourish kidney yin. Additionally, *Alisma orientale*, *Poria cocos*, and Moutan cortex are utilized to clear and purge the deficient fire in the liver and kidney, as well as to promote diuresis and percolate dampness. Consequently, this formula can be effectively applied to treat complex syndromes characterized by yin deficiency with fire hyperactivity and yin deficiency with damp-heat through modification by addition or subtraction of ingredients. It has demonstrated significant therapeutic efficacy and has been shown to improve symptoms in nephrotic syndrome caused by kidney yin deficiency and yin deficiency with fire hyperactivity [92].

Clinical studies have indicated that the addition of ZBDHP to conventional CWM treatment for DKD can effectively improve patients'blood glucose, blood lipid, and renal function levels in the short term, enhance clinical efficacy, and has not been associated with a significant increase in adverse reactions [93, 94]. Furthermore, the combination of ZBDHP and Irbesartan Tablets has exhibited good clinical efficacy in the treatment of DKD. It can improve renal function, reduce serum inflammatory factor levels, and has demonstrated high safety, suggesting potential value for clinical promotion and application [95].

### Turbid toxin internal accumulation pattern DKD

Suye Huanglian Decoction (SYHLD), which possesses the effects of promoting blood circulation, regulating qi movement, harmonizing the stomach, reducing nausea, and transforming dampness to eliminate turbidity, has been shown to significantly improve the uremic stage of chronic renal failure [96]. Studies have demonstrated that SYHLD is suitable for DKD caused by internal accumulation of damp-heat. It can reduce glycation stress-mediated renal fibrosis and improve DKD by downregulating the EGFR-p38 mitogen-activated protein kinase (MAPK) pathway [97]. Additionally, Taohe Chengqi Decoction (THCQD), which has the effects of breaking blood, removing blood stasis, purging heat, and expelling stasis, is also appropriate for DKD caused by blood stasis and excess syndrome [98]. Research indicates that THCQD modified with additional herbs can reduce the Scr levels in patients with stage V DKD, primarily through mechanisms such as anti-inflammation, oxidative phosphorylation, and mediating purine metabolism [99, 100].

### Blood Stasis Obstructing Collaterals Pattern DKD

Xuefu Zhuyu Decoction (XFZYD) is characterized by its effects of promoting blood circulation to remove blood stasis and regulating qi to relieve pain. It is suitable for DKD caused by blood stasis and qi stagnation. Studies have shown that the total effective rate of the patient group treated with XFZYD is significantly higher than that of the control group [101]. After treatment, indicators such as the urinary protein excretion rate and Scr levels in patients have been significantly improved, and the levels of TNF-α and MCP-1 in peripheral blood have been significantly reduced [102]. These findings suggest that XFZYD can effectively alleviate symptoms in patients with DKD, improve renal function, and has high clinical application value. Currently, research in this area primarily focuses on clinical applications. Another study, through the construction of a drug-target-disease network, has revealed that XFZYD may exert its effects through signaling pathways such as Janus kinase/signal transducer and activator of transcription (JAK-STAT) and TNF [103]. However, further in-depth research is still required to elucidate its specific mechanisms.

### Water-dampness flooding pattern DKD

Huangkui Capsule (HKC), derived from *Abelmoschus manihot*, is a contemporary botanical preparation. It possesses the properties of clearing damp-heat, detoxifying, and reducing edema, and is extensively utilized in the management of renal fibrosis in the early stages of DKD [104]. In clinical practice, HKC has been shown to exert significant diuretic and anti-edema effects in patients with the water-dampness flooding pattern of DKD, effectively alleviating symptoms of edema. Its mechanism of action may involve the regulation of systemic immune function, attenuation of inflammatory responses, and improvement of renal microcirculation, thereby reducing urinary protein leakage and protecting renal function [105]. A multi-center, double-blind, double-dummy, randomized controlled clinical trial demonstrated that HKC, in combination with angiotensin receptor blockers (ARB) or angiotensin-converting enzyme inhibitors (ACEI), significantly reduced key indicators such as the urinary albumin-creatinine ratio, 24-h urinary protein quantification, Scr, BUN. This combination therapy improved renal function, reduced proteinuria, delayed the progression of renal failure, and enhanced the quality of life of patients [106]. Additionally, studies have shown that HKC alleviates renal fibrosis through multiple pathways, including the regulation of oxidative stress and the p38MAPK/Akt signaling axis [107], inhibition of the Klotho-dependent TGF-β1/p38MAPK signaling pathway [108], activation of peroxisome proliferator-activated receptor alpha/gamma (PPARα/γ) to reduce endoplasmic reticulum stress [109], inhibition of NLRP3 inflammasome activation, and suppression of the TLR4/NF-κB signaling cascade [110]. Single-cell RNA sequencing of kidneys from db/db mice has elucidated the molecular mechanisms underlying the renoprotective effects of HKC. The flavonoid components of HKC directly interact with key receptors on renal cells, modulating downstream transcription factor activity, inhibiting T-cell-mediated inflammatory responses, and influencing transcriptional events in renal stromal cells associated with renal injury and proteinuria. These findings demonstrate the cell-type-specific renoprotective effects of HKC, providing robust scientific evidence for its application in the treatment of DKD [111].

### Spleen deficiency with dampness excess pattern DKD

In the treatment of DKD with the spleen-deficiency and damp-exuberance syndrome in TCM, the core therapeutic principles include invigorating the spleen and qi, resolving dampness and promoting diuresis, and clearing heat and dampness. Commonly used formulas include Yi Shen Hua Shi Granules (YSHSG) and Shen Ling Bai Zhu San (SLBZS), among others.

YSHSG are widely used in the treatment of various kidney diseases [112]. Numerous studies have confirmed that YSHSG can significantly enhance therapeutic efficacy and exhibit favorable medication safety when used as an adjuvant treatment for early-stage DKD [113, 114]. Further research suggests that its mechanism of action may involve regulating the"gut-kidney axis"and ameliorating podocyte damage induced by macrophage-derived exosomes [115, 116].

SLBZS, a classic TCM formula, can effectively optimize patient symptoms, increase urine output, and improve liver and kidney functions when used to treat edema in DKD through modification by addition or subtraction of ingredients, thereby enhancing the overall therapeutic effect [117]. Network pharmacology studies have identified that the effective active components in SLBZS, such as quercetin, nobiletin, and luteolin, can act on core targets such as TNF, tumor protein 53 (TP53), and STAT3 by regulating signaling pathways involved in oxidative stress and inflammatory responses, achieving multi-pathway and multi-target regulation of DKD (Table 1, at the end of the article) [118].

**Tabel 1:** TCM formulas with therapeutic effects on DKD

TCM pattern	TCM formulae	Compouds/consistuents		Effect	Specific mechanisms		References
Qi and Yin Deficiency with Blood Stasis Pattern	Shenqi Dihuang Decoction	*Panax ginseng* (Ren shen), Astragalus, *Rehmannia glutinosa*, Chinese yam, Dogwood fruit, *Alisma orientale, Poria cocos and* Moutan cortex		Regulate blood sugar level and reduce kidney damage	↓ACSL4/LPCAT3/ALOX15 axis and iron death		[72, 73]
	Keluoxin Capsule	*Astragalus membranaceus*, Glossy Privet Fruit (*Ligustrum lucidum*), Leech (Hirudo), Rhubarb (*Rheum palmatum*), Heterophylly Falsestarwort Root (*Pseudostellaria heterophylla*), Barbary Wolfberry Fruit (*Lycium barbarum*)		Relieve renal fibrosis injury	↓PERK-ATF4-CHOP signal pathway,↑Podocyte autophagy		[74, 77]
Spleen-Kidney Yang Deficiency Pattern	Zhenwu Decoction	Radix Aconiti Lateralis Preparata, *Poria cocos*, *Zingiber officinale* (Ginger), *Rhizoma Atractylodis Macrocephalae,Paeonia lactiflora*		Reduce urinary protein. Decrease the incidence of ESKD	↑Renal tubular Nrf2 and TFAM,↓Nrf2/HO-1/GPX4		[79–86]
	Jinkui Shenqi Pill	*Rehmannia glutinosa, Dioscorea opposita, Cornus officinalis, Alisma orientale, Poria cocos,* Moutan cortex*,* Cinnamomum cassia twig, and Radix Aconiti Lateralis Preparata		Reduce 24 h urinary protein excretion rate, decrease BUN, Scr	↓MHC II molecules and ↓antigen presentation of MHC II to CD4		[87, 88]
Liver-Kidney Yin Deficiency Pattern	Liuwei Dihuang Pill	Prepared *Rehmannia Root* (Shu Di Huang), *Cornus Officinalis* (Shan Zhu Yu), *Chinese Yam* (Shan Yao), *Alisma Orientalis* (Ze Xie), Moutan Bark (Mu Dan Pi), and *Poria Cocos* (Fu Ling)		Improve insulin resistance and reduce blood glucose	↓TGF -β, α- SMA protein, Smad2, and Smad3		[89–91]
Yin Deficiency with Dryness-Heat Pattern	Zhibai Dihuang Pill	*Anemarrhena asphodeloides,* Cortex *Phellodendri*, Prepared *Rehmannia Root* (Shu Di Huang), *Chinese Yam* (Shan Yao)*, Cornus Officinalis* (Shan Zhu Yu), *Alisma Orientalis* (Ze Xie), *Poria Cocos* (Fu Ling), and Moutan Bark (Mu Dan Pi)		Lowering blood sugar, regulating blood lipid and renal function	↓TNF-α, IL-6		[92–95]
Turbid Toxin Internal Accumulation Pattern	Suye Huanglian Decoction	*Perilla Leaf*(Su ye), *Coptis Rhizome*(Huang lian)		Decrease urea nitrogen, creatinine, endogenous creatinine clearance rate and 24 h urinary protein	↓EGFR-p38MAPK pathway		[96, 97]
	Taohe Chengqi Decoction		*Semen Persicae* (Taoren), *Radix et Rhizoma Rhei* (Dahuang), *Ramulus Cinnamomi* (Guizhi), *Radix Glycyrrhizae Praeparata cum Melle* (Zhigancao), *Natrii Sulfas* (Mangxiao)	Reduce FBG,24-h urinary protein quantification,	↓Oxidative phosphorylation damage		[98–100]
Blood Stasis Obstructing Collaterals Pattern	Xuefu Zhuyu Decoction		*Semen Persicae* (tao ren), *Flos Carthami* (hong hua), *Radix Angelicae Sinensis* (dang gui), *Radix Rehmanniae* (sheng di huang), *Rhizoma Chuanxiong* (chuan xiong), *Radix Paeoniae Rubra* (chi shao), *Radix Achyranthis Bidentatae* (niu xi), *Platycodon Grandiflorum* (jie geng), *Radix Bupleuri* (chai hu), *Fructus Aurantii* (zhi qiao), *and Radix Glycyrrhizae* (gan cao)	Reduce urinary protein excretion rate and Scr	↓JAK-STAT/TNF pathway, ↓TNF-α/MCP-1	[101, 102]	
Water-Dampness Flooding Pattern	Huangkui Capsule		*Abelmoschus manihot (L.) Medicus*	Relieve edema, improve renal microcirculation and reduce urinary protein excretion	↓Klotho-dependent TGF-β1/p38MAPK and NLRP3,↑PPARα/γ	[105–110]	
Spleen Deficiency with Dampness Excess Pattern	Yi Shen Hua Shi Granules		*Radix Astragali* (Huangqi), *Poria* (Fuling), *Rhizoma Dioscoreae* (Shanyao), *Rhizoma Alismatis* (Zexie), *Rhizoma Atractylodis Macrocephalae* (Baizhu), *Rhizoma Cimicifugae* (Shengma), *Radix Bupleuri* (Chaihu), *Herba Leonuri* (Yimucao), *Herba Epimedii* (Yinyanghuo), *Psoralea corylifolia* (Buguzhi), *Fructus Rosae Laevigatae* (Jinyingzi), *Radix Rehmanniae Recens* (Shengdihuang), *Radix Glycyrrhizae* (Gancao)	Lowering blood pressure and reduce renal fibrosis	↓Podocyte damage induced by exosomes derived from macrophages and regulate gut-kidney axis	[113–116]	
	Shenlingbaizhu Powder		*Radix Ginseng* (Renshen), *Atractylodis Macrocephalae Rhizoma* (Baizhu), *Poria* (Fuling), *Glycyrrhrizae Radix et Rhizoma Praeparata cum Melle* (Zhigancao), *Dioscoreae Rhizoma* (Shanyao), *Nelumbinis Semen* (Lianzi) *Dolichoris Lablab Semen* (Baidou), *Coicis Semen* (Yiyiren), *Amomi Fructus* (Sharen), *Platycodonis Radix* (Jiegeng)	Relieve edema, increase urination, and improve liver and kidney function	↓TNF/tp53/STAT3	[117, 118]	

**Fig. 2:**
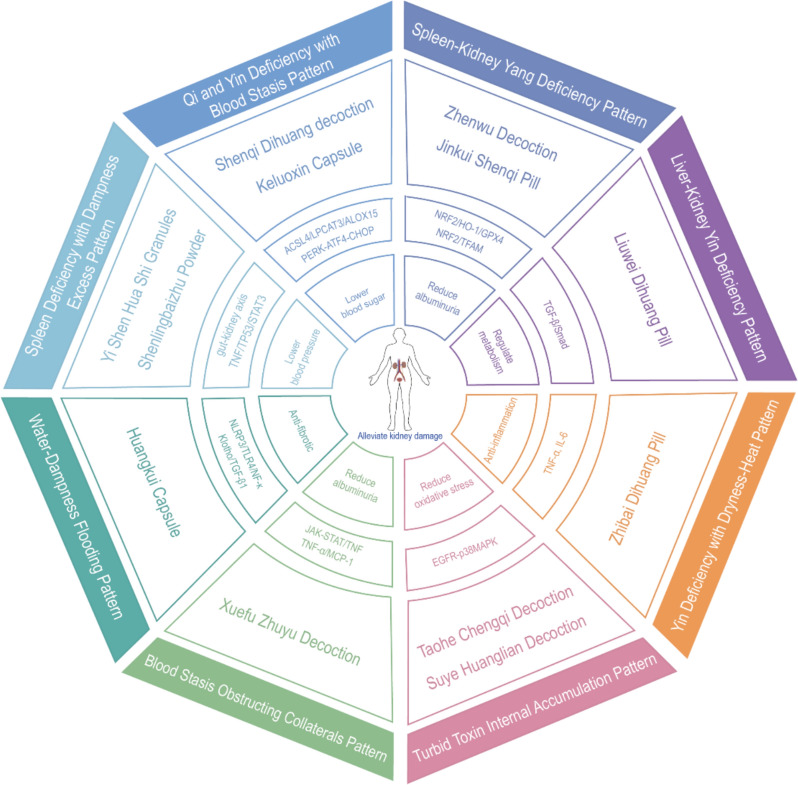
TCM syndromes-prescriptions-mechanisms map in DKD

The mechanisms underlying the treatment of DKD with TCM are multifaceted, encompassing several critical aspects, including the regulation of metabolic disorders, improvement of renal microcirculation, counteraction of oxidative stress, and anti-inflammatory effects. These mechanisms act in concert, providing comprehensive theoretical support for the holistic treatment of DKD (Fig. 2).

While TCM formulas demonstrate clinical efficacy through the synergistic action of multiple components, recent advances in pharmacological research have begun to elucidate the molecular mechanisms of their bioactive constituents. The following section highlights key TCM-derived compounds and their targeted actions against pathways related to DKD.

## Research progress of TCM active ingredients'mechanisms in treating DKD

Emerging evidence underscores the unique advantages of TCM in diabetes management through its characteristic"multi-component, multi-target, multi-pathway"synergistic mechanisms. Recent advancements in mechanistic studies have revealed that bioactive constituents of TCM exert therapeutic effects by comprehensively modulating critical pathological processes, including suppression of oxidative stress, amelioration of inflammatory responses and fibrotic progression, metabolic homeostasis regulation, podocyte preservation, and epigenetic modulation. These multi-dimensional pharmacological actions demonstrate superior therapeutic potential in addressing the complex pathogenesis of diabetes mellitus compared to single-target approaches, providing novel insights for developing integrative treatment strategies against this multifactorial metabolic disorder.

### Oxidative stress suppression and antioxidant defense

Oxidative stress plays a crucial role in the development and progression of DKD. Excessive ROS can damage renal cells, leading to renal dysfunction. Several TCM components and extracts have demonstrated significant antioxidant properties in this context.

Isoliquiritigenin, a flavonoid derived from Glycyrrhiza glabra, has been shown to protect human renal proximal tubular epithelial cells (HK-2) under high-glucose conditions. It enhances cell viability by increasing the levels of glutathione, superoxide dismutase 2, and glutathione peroxidase 1, which are important antioxidant enzymes. Simultaneously, isoliquiritigenin reduces ROS production. In streptozotocin (STZ)-induced diabetic rats, isoliquiritigenin not only improves renal function parameters but also inhibits oxidative stress, suggesting its potential as a therapeutic agent for DKD [119].

Hyperoside, a glycoside derivative of quercetin, contributes to renal protection in diabetic conditions. In diabetic mice, it reduces early-stage albuminuria and GBM damage by decreasing the expression of podocyte heparanase. Additionally, hyperoside inhibits renal inflammation through modulating macrophage polarization. This dual function of hyperoside, both in reducing oxidative stress-related damage and inflammation, highlights its importance in the treatment of DKD [120–122]. Pharmacokinetic studies show that hyperoside primarily accumulates in the kidneys. When combined with other drugs, its metabolism time may be prolonged, thereby increasing the risk of renal accumulation. Animal studies indicate that long-term high-dose use of hyperoside may lead to renal tubular damage and decreased GFR [123]. However, there is insufficient evidence on the nephrotoxicity of long-term low-dose use of hyperoside, and human clinical data are lacking to support it [124].

Salidroside, another TCM component, inhibits oxidative stress and inflammation through the Akt/glycogen synthase kinase-3 (GSK-3) signaling pathway. Activation of this pathway leads to a series of cellular responses that protect against the harmful effects of oxidative stress. In rats with STZ-induced DKD, salidroside effectively protects the kidneys, indicating its potential in preventing disease progression [125].

Goji leaf extract exerts its beneficial effects on DKD mainly by inhibiting hyperglycemia-mediated renal oxidative stress and inflammatory responses. High blood glucose levels in diabetes can trigger the overproduction of ROS, which in turn activates inflammatory pathways. Goji leaf extract interrupts this vicious cycle, thereby ameliorating DKD [126]. Garlic extract shows multiple beneficial effects in diabetic rat kidneys. It reduces glucose, uric acid, and urea levels, which are often elevated in diabetic conditions. Moreover, garlic extract alleviates oxidative stress, further protecting the kidneys from damage. These combined effects of garlic extract suggest its potential in the management of DKD [127].

### Inflammation and fibrosis modulation

Inflammation and fibrosis are two key pathological processes in DKD. Chronic inflammation can lead to tissue damage and the activation of fibrotic pathways, resulting in the progressive loss of renal function. TCM components and extracts have been found to modulate these processes effectively.

Acteoside, a flavonoid compound predominantly found in Echinacea purpurea, alleviates kidney injury in diabetic db/db mice and high-glucose-induced HK-2 cells. It does so by inhibiting the nicotinamide adenine dinucleotide phosphate hydrogen (NADPH) oxidase-TGF-β/Smad signaling pathway, which is a key regulator of oxidative stress and fibrosis. Additionally, acteoside regulates disrupted metabolic pathways, such as lipid metabolism. In unilateral nephrectomy combined with STZ-induced DKD rat models, acteoside further demonstrates its renoprotective effects by suppressing the expression of pyroptosis markers, including NLRP3, Caspase-1, and IL-1β, via the PI3K/AKT/NF-κB pathway [128, 129]. Baicalin, a flavonoid with diverse biological activities, plays a crucial role in preventing renal fibrosis. It upregulates the expression of miR-124, which can inhibit the TLR4/NF-κB signaling pathway, a major pathway involved in inflammation and fibrosis. Moreover, baicalin modulates the methylation status of the Klotho gene promoter region, which helps to mitigate diabetic acidosis-induced renal injury [130, 131]. Wogonin reduces renal inflammation and fibrosis through multiple mechanisms. It upregulates suppressor of cytokine signaling 3 (SOCS3), which in turn inhibits the TLR4, JAK/STAT, and TGF-β1/Smad3 pathways. These pathways are central to the inflammatory and fibrotic processes in the kidney. Additionally, wogonin mitigates tubular interstitial fibrosis via PI3K/Akt/NF-κB-mediated autophagy, highlighting its comprehensive effects on renal protection [132–134].

Ligustrazine, a pyrazine alkaloid compound extracted from the rhizomes of Ligusticum wallichii, alleviates inflammation and renal fibrosis through multiple actions. It suppresses the activation of NF-κB, a key transcription factor in the inflammatory response. It also inhibits the proliferation of mesangial cells, which is an important event in the development of renal fibrosis. Furthermore, ligustrazine promotes autophagy, which can help remove damaged organelles and proteins, thus protecting the kidney from further damage [135]. Caution is warranted regarding potential drug-drug interactions involving ligustrazine, which may increase the risk of adverse effects or compromise therapeutic efficacy. Due to the paucity of safety data on ligustrazine use in pregnant and lactating women, extreme caution should be exercised when administering this agent to these populations. Ligusticum chuanxiong extract (EEL) demonstrates significant efficacy in suppressing oxidative stress and inflammation. By reducing the production of inflammatory mediators and ROS, EEL effectively alleviates the degree of glomerular sclerosis and fibrosis, contributing to the preservation of renal function [136]. *Prunella vulgaris* extract (APV) alleviates glomerular fibrosis and inflammation by suppressing the TGF-β signaling pathway. TGF-β is a well-known pro-fibrotic factor, and its inhibition by APV can effectively slow down the progression of renal fibrosis and inflammation in DKD [137].

### Mitochondrial and endoplasmic reticulum (ER) stress regulation

Mitochondrial and ER stress are closely associated with the pathogenesis of DKD. Dysfunction in these organelles can lead to cell apoptosis and the impairment of renal function. TCM components have shown the ability to regulate these stress responses.

Berberine protects podocytes, which are crucial cells in the glomerulus, by inhibiting dynamin-related protein 1 (Drp1)-mediated mitochondrial fission and dysfunction. Drp1-mediated mitochondrial fission can lead to mitochondrial fragmentation and loss of normal function [138]. Berberine enhances mitochondrial energy homeostasis via the activation of PGC-1α. This activation promotes fatty acid oxidation and mitochondrial biogenesis, thereby reversing metabolic disorders and glomerulosclerosis in DKD [139]. Berberine demonstrates minimal toxicity at conventional doses and confers significant clinical benefits without major adverse effects. Mild gastrointestinal discomfort (e.g., nausea, diarrhea) may be observed in some patients, with an incidence comparable to that of standard treatments. Systematic evaluations have consistently confirmed its favorable safety profile, with no serious adverse events reported [140, 141].

Astragaloside IV (AS-IV), a principal bioactive constituent of Astragalus membranaceus, effectively inhibits ER stress-induced apoptosis in renal tubular cells. This action is mediated through the IRE-1α/NF-κB/NLRP3 axis, a complex signaling pathway implicated in the ER stress response and inflammatory processes. Furthermore, AS-IV attenuates NLRP3 inflammasome-mediated podocyte injury, thereby safeguarding the structural integrity of the glomerulus and preserving renal function [142–144]. A recent study employing multi-omics analysis, molecular docking, and molecular dynamics simulations has elucidated that AS-IV can mitigate ferroptosis in renal tubular epithelial cells by downregulating the hypoxia-inducible factor 1α (HIF-1α)/HO-1 signaling pathway [145]. Current research indicates that AS-IV exerts minimal effects on hepatic and renal function at conventional doses, with no significant toxicity observed. However, given its potential for reproductive toxicity during pregnancy and possible drug interactions, caution is advised in clinical applications. Further investigation into its long-term toxicity and safety profile is warranted [146].

Resveratrol, a naturally occurring polyphenolic compound, attenuates oxidative stress-mediated podocyte apoptosis through the activation of AMPK. AMPK serves as a pivotal regulator of cellular energy metabolism, and its activation by resveratrol confers protection against oxidative stress-induced cellular damage. In db/db mice, resveratrol has also been shown to modulate autophagy by inhibiting miR-383-5p, a microRNA involved in the regulation of autophagy-related genes [147, 148]. *Ranunculus ternatus* Extract (RTT) reduces the expression levels of TNF-α, SET, and MYND Domain Containing Protein 2 (SMYD2). TNF-α, a pro-inflammatory cytokine, contributes to inflammation-related mitochondrial dysfunction in renal cells. Its downregulation by RTT can thus alleviate such dysfunction. SMYD2 is also implicated in mitochondrial function, and its reduced expression by RTT can enhance mitochondrial function in the kidneys of STZ-induced diabetic mice [149]. Resveratrol is generally considered safe at conventional doses. However, high-dose administration may induce gastrointestinal discomfort and hepatic dysfunction. In vitro studies have demonstrated its inhibitory effect on human platelet aggregation, and its concurrent use with anticoagulants, antiplatelet agents, or nonsteroidal anti-inflammatory drugs may increase the risk of bruising and bleeding. Special populations, including pregnant women, children, patients with hemorrhagic disorders, and individuals with hormone-sensitive conditions, should exercise particular caution due to the potential adverse effects. Further research is essential to investigate long-term toxicity profiles and to establish comprehensive safety guidelines for rational clinical utilization [150, 151].

### Metabolic pathway regulation

Diabetes is characterized by metabolic abnormalities, and the regulation of metabolic pathways is essential for the treatment of DKD. TCM components and extracts have demonstrated the ability to modulate various metabolic pathways, thereby improving the pathological conditions associated with diabetes and its complications.

Quercetin and Hesperidin, two flavonoids, have been shown to ameliorate DKD in T2DM models through multiple mechanisms, including antioxidant, anti-inflammatory, and anti-fibrotic actions. These compounds also modulate lipid metabolism, which is frequently dysregulated in diabetes. In STZ-induced diabetic rats, administration of Quercetin and Hesperidin results in a reduction of renal injury markers, indicating their beneficial effects on kidney function [152–155]. The ethanolic extract from the rhizome of *Polygonum aviculare* (ER-PA) has been shown to improve insulin resistance, a key feature of diabetes. By regulating metabolic pathways, ER-PA inhibits the progression of nephritis in DKD. Insulin resistance is associated with abnormal glucose and lipid metabolism, and the improvement of insulin resistance by ER-PA can help correct these metabolic disorders and slow the progression of DKD [156]. Currently, there is no direct evidence explicitly supporting or negating the clinical toxicity risk of ER-PA. Given the lack of dedicated toxicity studies on this extract, it is recommended to monitor individual variations during use and remain vigilant for potential allergic reactions. Corn Silk extract (CS) has demonstrated hypoglycemic and antioxidant effects. In high-glucose-treated mesangial cells, CS suppresses the production of Collagen Type IV, Fibronectin, and IL-6. These proteins are involved in ECM remodeling and inflammation in the kidney. The suppression of their production by CS suggests its potential in protecting the kidneys from damage caused by high-glucose-induced metabolic disorders [157].

### Podocyte protection and glomerular integrity

Podocytes are critical for maintaining the normal function of the glomerulus, and their damage is a key event in the development of DKD. TCM components and extracts have demonstrated the ability to protect podocytes and preserve glomerular integrity. Puerarin, an isoflavone derived from Pueraria lobata, activates the cyclic adenosine monophosphate/protein kinase A/cyclic AMP response element-binding protein (cAMP/PKA/CREB) signaling pathway. This activation leads to a decrease in high-glucose-induced podocyte apoptosis. By promoting CREB phosphorylation, puerarin regulates the expression of anti-apoptotic genes, thereby protecting podocytes from injury and ameliorating diabetic kidney damage [158]. Curcumin protects podocytes through multiple mechanisms. It inhibits receptor-interacting serine/threonine-protein kinase 3 (RIPK3)-dependent pathways, which are involved in necroptosis, a form of programmed cell death [159]. Curcumin also restores the balance of nephrin, VEGF, and TGF-β, which are important factors for maintaining podocyte function. Additionally, curcumin modulates the Wnt signaling pathway to reduce oxidative stress, further protecting podocytes from high-glucose-induced injury [160, 161].

Phillyrin activates the PI3K/Akt/GSK-3β signaling pathway, which plays a crucial role in cell survival and proliferation. In DKD mice, phillyrin reduces renal damage primarily by suppressing oxidative stress and apoptosis. The activation of this pathway can promote the phosphorylation of downstream targets, leading to the inhibition of apoptosis and the protection of renal cells, including podocytes [162]. *Salvia miltiorrhiza* extract (EASM) treatment reduced albuminuria, improved renal function, and alleviated pathological alterations within the glomerulus in STZ-induced mice. Additionally, EASM inhibited high glucose-induced ROS in mouse mesangial cells and enhanced the expression of Nrf2, HO-1, and quinone oxidoreductase 1 (NQO1) while decreasing Keap1 expression, both in vitro and in vivo. These findings suggest that EASM may alleviate DKD progression through Nrf2 activation. [163].

### Epigenetic and autophagy regulation

Epigenetic modifications and autophagy are integral to the pathogenesis of DKD. TCM components have demonstrated the capacity to modulate these processes, thereby exerting protective effects on the kidneys.

Apigenin, a naturally occurring flavonoid, inhibits the mitogen-activated protein kinase (MAPK) pathway, which is implicated in various cellular responses, including oxidative stress, inflammation, and fibrosis. In STZ-induced diabetic rats, apigenin attenuates renal dysfunction, oxidative stress, and fibrosis by inhibiting this pathway. The suppression of the MAPK pathway leads to the downregulation of pro-inflammatory and pro-fibrotic genes, thereby protecting the kidneys from damage [164]. Apigenin is generally considered safe at conventional doses; however, high-dose administration may induce adverse reactions such as gastrointestinal discomfort, sedation, and muscle relaxation. The potential risks associated with high-dose administration should be carefully evaluated prior to clinical use [165]. Wogonin, a flavone derived from *Scutellaria baicalensis*, reduces podocyte damage by modulating the crosstalk between autophagy and apoptosis, mediated by B-cell lymphoma-2 (Bcl-2). Bcl-2 is a key regulator of both apoptosis and autophagy. Wogonin modulates Bcl-2 function, thereby promoting autophagy and inhibiting apoptosis, which in turn protects podocytes from damage [166]. *Desmodium caudatum* extract (DCE) mitigates renal fibrosis by reversing the EMT. This effect is achieved through the modulation of Klotho promoter methylation and the enhancement of antioxidant enzyme activity. Klotho is a critical protein for maintaining renal function, and the regulation of its promoter methylation by DCE can modulate its expression, thereby protecting the kidneys from fibrosis [167].

### Multi-target plant extracts.

Certain plant extracts exhibit multifaceted effects on DKD, with their mechanisms of action often being intricate and encompassing multiple pathways. For instance, Bamboo Leaf extract demonstrates a distinct regulatory influence on pivotal signaling molecules. Specifically, it downregulates phosphorylated GSK-3β and B-cell lymphoma-2-associated X protein (BAX), while simultaneously upregulating phosphorylated PKB. These alterations in protein expression can impact a variety of cellular processes, including metabolism, apoptosis, and cell survival, thereby contributing to the amelioration of renal function in diabetic rats [168].

*Psoralea corylifolia* extract (PCS) inhibits fibrosis and apoptosis through synergistic pathways that remain to be fully elucidated. While the precise mechanisms are not yet fully understood, the combined effects of PCS on these two critical pathological processes suggest its potential utility in the treatment of DKD. Further studies are warranted to elucidate the specific molecular mechanisms underlying its actions (Table 2, at the end of the article) [169]. Emerging evidence indicates that PCS may induce hepatotoxicity via metabolic activation pathways, although nephrotoxicity has not been linked to psoralen. Current toxicity assessments primarily focus on isolated constituents, potentially overlooking synergistic interactions that could modulate the extract’s toxicological profile [170].

**Table 2:** Action mechanisms of active ingredients in TCM for DKD

Mechanism overview	Specific mechanisms	Representative active ingredients	References
Oxidative stress suppression and antioxidant defense	Boost antioxidant enzymes, cut down ROS	Isoliquiritigenin	[119]
	Lower heparanase, regulate macrophages	Hyperoside	[120–122]
	Inhibit via Akt/GSK-3β pathway	Salidroside	[125]
	Block hyperglycemia- driven stress and inflammation	Goji Leaf Extract	[126]
	Reduce metabolites, ease oxidative stress	Garlic Extract	[127]
Inflammation and fibrosis modulation	Inhibit NADPH-TGF-β/Smad, regulate metabolism, suppress pyroptosis	Acteoside	[128, 129]
	Upregulate miR-124, inhibit TLR4/NF-κB, modulate Klotho methylation	Baicalin	[130, 131]
	Upregulate SOCS3, inhibit key pathways, mitigate fibrosis via autophagy	Wogonin	[132–134]
	Suppress NF-κB, inhibit mesangial cells, promote autophagy	Ligustrazine	[135]
	Reduce mediators and ROS, ease glomerular issues	Ligusticum chuanxiong Extract	[136]
	Inhibit TGF-β pathway	Prunella vulgaris Extract	[137]
Mitochondrial and endoplasmic reticulum stress regulation	Inhibit Drp1, activate PGC-1α, promote fatty acid and mitochondrial functions	Berberine	[138, 139]
	Inhibit apoptosis via IRE-1α axis, combat podocyte injury	Astragaloside IV	[142–144]
	Activate AMPK, cut apoptosis, regulate autophagy	Resveratrol	[147, 148]
	Reduce TNF-α and SMYD2, ease mitochondrial dysfunction	Ranunculus ternatus Extract	[149]
Metabolic pathway regulation	Antioxidant, anti-inflammatory, anti- fibrotic, regulate lipids	Quercetin, Hesperidin	[152–155]
	Improve insulin resistance, regulate pathways	The ethanolic extract from rhizome of Polygoni avicularis	[156]
	Hypoglycemic, antioxidant, suppress matrix and inflammatory proteins	Corn Silk Extract	[157]
Podocyte protection and glomerular integrity	Activate cAMP/PKA/CREB, cut apoptosis, regulate anti- apoptotic genes	Puerarin	[158]
	Inhibit RIPK3, restore balance, regulate Wnt	Curcumin	[159–161]
	Activate PI3K/Akt/GSK −3β, inhibit stress and apoptosis	Phillyrin	[162]
	Reduce albuminuria, activate Nrf2, inhibit ROS	Salvia miltiorrhiza Extract	[163]
Epigenetic and autophagy regulation	Inhibit MAPK, down–regulate pro- inflammatory and- fibrotic genes	Apigenin	[164]
	Regulate Bcl-2-mediated autophagy and apoptosis	Wogonin	[166]
	Reverse EMT, modulate Klotho methylation, boost antioxidant enzymes	Desmodium caudatum Extract	[167]
Multi-target plant extracts	Downregulate P-GSK −3β and BAX, upregulate P-AKT	Bamboo Leaf Extract	[168]
	Downregulate PARP, reduce fibrosis-related mRNA, inhibit fibrosis and apoptosis	Psoralea corylifolia Extract	[169]

The cumulative evidence highlights the potential of TCM to bridge the gap between holistic regulation and molecular precision in the management of DKD. However, significant challenges persist in translating these findings into standardized clinical practice. The subsequent discussion synthesizes the current state of knowledge and delineates future directions for TCM research.

## Discussion and outlook

The innovative potential of TCM in managing DKD is characterized by its spatiotemporal therapeutic framework, which dynamically adapts to the heterogeneity of the disease. This multi-target approach synergistically modulates genetic, metabolic, and inflammatory pathways through stage-specific interventions aligned with the progression of DKD. The dynamic staging model is particularly promising, especially in the early stages (G1-2), where SQDHD inhibits ferroptosis by modulating the ACSL4/LPCAT3/ALOX15 axis, effectively addressing glomerular hyperfiltration and oxidative stress. Its “Qi-invigorating and Yin-nourishing” effects correlate with the clinical stabilization of eGFR and reduced urinary microalbumin levels. In moderate stages (G3), HKC suppresses TGF-β/Smad signaling and NLRP3 inflammasome activation, synergistically improving metabolic-inflammatory imbalances and delaying tubulointerstitial fibrosis. For advanced stages (G4-5), the combination of Dahuang Fuzi Decoction with colon dialysis targets the gut-kidney axis, reducing the accumulation of uremic toxins and improving renal anemia. This is achieved through the"detoxification and collateral-dredging"mechanism, as evidenced by decreased levels of serum urea nitrogen and creatinine. Clinical studies have confirmed that integrating this staged strategy with RAAS inhibitors synergistically reduces 24-h urinary protein excretion while maintaining renal function, thereby exemplifying TCM’s integrative advantage in"systemic regulation and targeted repair".

However, the translation of TCM into mainstream clinical practice presents significant challenges. Standardization remains a critical barrier, as the variability in bioactive compounds across batches can compromise the reproducibility of therapeutic effects. Pharmacokinetic studies are urgently needed to clarify absorption and distribution patterns, particularly for components with potential nephrotoxicity like aristolochic acid derivatives. While the integration of TCM with molecular-targeted therapies or interventions that modulate the gut-kidney axis holds promise, achieving such synergy necessitates robust collaboration among fields including pharmacology, nephrology, and data science. Large-scale randomized controlled trials with long-term follow-ups, repeatedly emphasized in recent systematic reviews, remain essential for validating efficacy and safety.

To address these challenges, future research should focus on integrating TCM with modern diagnostic and therapeutic approaches. By leveraging advanced analytical techniques such as single-cell transcriptomics and spatial multi-omics, we can gain a deeper understanding of how TCM components interact with renal tissues. This will help to substantiate the “multi-target” effects of TCM and provide a more precise mechanism of action. Additionally, establishing standardized protocols for TCM practice, including terminology and quality control, is essential for its broader acceptance and application globally. Through collaborative efforts across disciplines, TCM can become an integral part of personalized DKD treatment plans. This integration will validate TCM's comprehensive approach and enhance its contribution to contemporary healthcare, offering patients a more holistic and evidence-based treatment option.

## Data Availability

Data availability is not applicable to this article as no new data were created or analyzed in this study.

## Abbreviations

DKD: Diabetic kidney disease
TCM: Traditional Chinese medicine
CKD: Chronic kidney disease
ESKD: End-stage kidney disease
GFR: Glomerular filtration rate
GBM: Glomerular basement membrane
ECM: Extracellular matrix
RAAS: Renin–angiotensin–aldosterone system
SGLT2: Sodium-glucose cotransporter 2
DPP-4: Dipeptidyl peptidase-4
PKC: Protein kinase C
MRAs: Mineralocorticoid receptor antagonists
ETR: Endothelin receptor
SNPs: Single nucleotide polymorphisms
COL4A3: Collagen type IV alpha 3 chain
miRNAs: MicroRNAs
lncRNAs: Long non-coding RNAs
TGF-β: Transforming growth factor-beta
PTEN: Phosphate and tension homology deleted on chromosome ten
PI3K/Akt: Phosphatidylinositol 3-Kinase/Protein Kinase B
NF-κB: Nuclear factor kappa-B
PGC-1α: Peroxisome proliferator-activated receptor gamma coactivator, 1-alpha
MALAT1: Metastasis associated lung adenocarcinoma transcript 1
PVT1: Plasmacytoma variant translocation 1
ANRIL: Antisense noncoding RNA gene at the INK4 locus
Erbb4-IR: Erb-B2 receptor tyrosine kinase 4-immunoreactivity
AGEs: Advanced glycation end-products
VEGF: Vascular endothelial growth factor
AR: Aldose reductase
ROS: Reactive oxygen species
EMT: Epithelial–mesenchymal transition
NLRP3: Nucleotide-binding oligomerization domain-like receptor family pyrin domain-containing 3
IL-1β: Interleukin-1 beta
CRP: C-reactive protein
NR4A1: Nuclear receptor subfamily 4 group A member 1
IRE1α: Inositol requiring enzyme 1 alpha
sXBP1: Spliced X-box binding protein 1
CTGF: Connective Tissue Growth Factor
MCP-1: Monocyte chemoattractant protein-1
mTOR: Mammalian target of rapamycin
AMPK: AMP-activated protein kinase
SIRT1: Silent information regulator T1
mTORC1: Mammalian target of rapamycin complex 1
TFEB: Transcription factor EB
TNF-α: Tumor necrosis factor-alpha
GECs: Glomerular endothelial cells
GSDMD: Gasdermin D
TLR4: Toll-like receptor 4
SQDHD: Shenqi Dihuang Decoction
ACSL4: Acyl-CoA Synthetase Long Chain Family Member 4
LPCAT3: Lysophosphatidylcholine Acyltransferase 3
ALOX15: Arachidonate 15-Lipoxygenase
RCTs: Randomized controlled trials
BUN: Blood urea nitrogen
Scr: Serum creatinine
KLXC: Keluoxin Capsule
PERK: Protein kinase R-like endoplasmic reticulum kinase
ATF4: Activating transcription factor 4
CHOP: DNA damage-inducible transcript 3
ZWD: Zhenwu Decoction
CWM: Conventional Western medicine
Nrf2: Nuclear factor erythroid 2-related factor 2
TFAM: Transcription factor A
HO-1: Heme oxygenase-1
GPX4: Glutathione peroxidase 4
JGSQW: Jingui Shenqi Pill
MHC class II: Major Histocompatibility Complex Class II
LWDHP: Liuwei Dihuang Pill
α-SMA: Alpha-Smooth Muscle Actin
ZBDHP: Zhibai Dihuang Pill
SYHLD: Suye Huanglian Decoction
MAPK: Mitogen-activated protein kinase
THCQD: Taohe Chengqi Decoction
XFZYD: Xuefu Zhuyu Decoction
JAK-STAT: Janus kinase/signal transducer and activator of transcription
HKC: Huangkui Capsule
ARB: Angiotensin receptor blockers
ACEI: Angiotensin-converting enzyme inhibitors
PPARα/γ: Peroxisome proliferator-activated receptor alpha/gamma
YSHSG: Yi Shen Hua Shi Granules
SLBZS: Shen Ling Bai Zhu San
TP53: Tumor protein 53
STZ: Streptozotocin
HK-2: Human kidney 2
NADPH: Nicotinamide adenine dinucleotide phosphate hydrogen
SOCS3: Suppressor of cytokine signaling 3
EEL: Ligusticum chuanxiong Extract
APV: Prunella vulgaris Extract
ER: Endoplasmic reticulum
Drp1: Dynamin-related protein 1
AS-IV: Astragaloside IV
HIF-1α: Hypoxia-inducible factor 1 alpha
RTT: Ranunculus ternatus Extract
SMYD2: SET and MYND Domain Containing Protein 2
ER-PA: Ethanolic extract from rhizome of Polygoni avicularis
CS: Corn Silk Extract
cAMP: Cyclic adenosine monophosphate
PKA: Protein kinase A
CREB: Cyclic AMP response element-binding protein
RIPK3: Threonine-protein kinase 3
EASM: Salvia miltiorrhiza Extract
NQO1: Quinone oxidoreductase 1
DCE: Desmodium caudatum Extract
Bcl-2: B-cell lymphoma-2
GSK-3: Glycogen synthase kinase-3
BAX: B-cell lymphoma-2-associated X protein
PCS: Psoralea corylifolia Extract
